# Activity of sEH and Oxidant Status during Systemic Bovine Coliform Mastitis

**DOI:** 10.3390/antiox10050812

**Published:** 2021-05-20

**Authors:** Vengai Mavangira, Matthew J. Kuhn, Angel Abuelo, Christophe Morisseau, Bruce D. Hammock, Lorraine M. Sordillo

**Affiliations:** 1 Department of Large Animal Clinical Sciences, College of Veterinary Medicine, Michigan State University, East Lansing, MI 48824, USA; kuhnmat2@gmail.com (M.J.K.); abuelo@msu.edu (A.A.); sordillo@msu.edu (L.M.S.); 2 Department of Entomology and Nematology, University of California Davis, Davis, CA 95616, USA; chmorisseau@ucdavis.edu (C.M.); bdhammock@ucdavis.edu (B.D.H.); 3 U.C. Davis Comprehensive Cancer Center, University of California Davis, Davis, CA 95616, USA

**Keywords:** cytochrome P450, inflammation, mastitis, oxidative stress, oxylipids, soluble epoxide hydrolase

## Abstract

Bovine coliform mastitis presents treatment challenges because of systemic inflammation and oxidative stress. Soluble epoxide hydrolase (sEH) is a promising therapeutic target in conditions characterized by inflammation and oxidative stress but has not been evaluated in cattle. We compared sEH activity and oxidant status in healthy Holstein dairy cows to those with systemic coliform mastitis (*n* = 5/group) using complementary approaches. First, the activity of sEH on [^3^H]-*trans*-diphenyl-propene oxide (tDPPO) was assessed ex vivo using tissue homogenates (mammary, liver, and kidney). Second, the concentrations of sEH substrates and metabolites in plasma, milk, and urine were determined as an index of in vivo sEH activity. Oxidant status was assessed in serum and milk. Data were analyzed by non-parametric methods. Metabolism of tDPPO was greater in mammary tissues from cows with coliform mastitis compared to controls. In contrast, ratios of sEH substrates and metabolites predicted lower sEH activity in cows with coliform mastitis than controls. Milk oxidant status showed greater prooxidant levels in coliform mastitis cows. Cows with coliform mastitis exhibit increased sEH activity in mammary tissue; at the same time, milk oxidant status is increased. Future studies should characterize sEH activity and oxidant status patterns and explore therapies targeting sEH during coliform mastitis.

## 1. Introduction

Bovine mastitis continues to substantially hamper dairy producers economically due to decreased milk production, poor milk quality, and increased veterinary and labor costs associated with infection [1]. In addition, systemic infections resulting from mastitis caused by Gram-negative bacteria compromise animal welfare and may result in death. Current therapies for coliform mastitis improve clinical signs in some cases; however, mortality or culling rates in systemically ill animals are not significantly different between treated and untreated cows [2,3,4]. The poor effectiveness of standard therapies may be linked to the severe inflammation, and importantly, the concurrent oxidative stress that accompanies coliform mastitis [5]. As such, there remains a major need to identify more effective therapies to improve treatment outcomes, such as those targeting the occurrence and severity of oxidative stress.

The oxygenation of polyunsaturated fatty acid (PUFA) during acute coliform mastitis results in the formation of potent inflammatory mediators, known as oxylipids, that contribute to ROS production and oxidative stress [6,7]. The oxygenation of PUFA to oxylipids is mediated nonenzymatically by reactive oxygen species and enzymatically by pathways including cyclooxygenases (COX), lipoxygenases, and the cytochrome P (CYP) 450 [8]. Oxylipids from the cyclooxygenase 2 (COX2) pathway are the most described and were identified in earlier studies as being overproduced during bovine coliform mastitis [9]. In addition to the limited treatment success of standard therapies, the use of nonsteroidal anti-inflammatory drugs (NSAIDs) is also complicated by their propensity to induce side effects, including renal and gastric toxicity [10,11]. Our recent studies revealed that increased production of oxylipids from the CYP450 pathway and soluble epoxide hydrolase (sEH) enzymes represented the most changes in oxylipid production during systemic coliform mastitis [7]. Several mammalian models of acute inflammation, similar to acute coliform mastitis, showed CYP450 and sEH pathways as beneficial targets for therapy [12]; the relevance of this pathway in bovine coliform mastitis, however, is unknown.

The epoxygenation of PUFA by the CYP450 pathway generates oxylipids known as epoxy fatty acids that regulate inflammation and mediate oxidative stress [13]. Epoxy fatty acids are hydrated by sEH to vicinal diols decreasing the anti-inflammatory and antioxidative properties of the epoxy fatty acids [14]. Vicinal diols can contribute to detrimental inflammation and oxidative stress responses [15]. Thus, inhibition of sEH, whose activity is enhanced during inflammation and oxidative stress in other animal species, [16] may improve treatment responses by limiting inflammation and oxidative stress during coliform mastitis. The activity of sEH in cattle with acute coliform mastitis has not been evaluated. The hypothesis of this study was that sEH activity increases during acute bovine coliform mastitis. Our study aimed to determine sEH activity at the site of infection, mammary tissue, and liver and kidneys because of systemic inflammation. We also determined the changes in oxidant status to verify changes supportive of oxidative stress during systemic coliform mastitis.

## 2. Materials and Methods

Holstein dairy cows diagnosed with systemic coliform mastitis and healthy controls (*n* = 5/group) were enrolled in this case-control study. Systemic clinical disease was judged by the presence of clinical signs, including hyperthermia, tachycardia, tachypnea, episcleral injection, moderate to systemic dehydration, in addition to local mammary gland inflammation. Systemic coliform mastitis was based on at least 2 clinical signs and a positive culture of *Escherichia coli* (*E. coli*) in milk from infected mammary glands. *Escherichia coli* was identified based on typical colony characteristics and growth on MacConkey agar which is selective for coliform bacteria based on National Mastitis Guidelines [17]. Bacterial growth was considered positive when there were greater than 3 colony forming units (CFU) per mL. Control cows were selected by matching lactation numbers and days from calving. Cows in the control group had no overt clinical signs and composite milk samples were negative on bacterial culture (<3 CFU/mL). At the time of sample collection, all mastitis cows had been treated with up to 2 doses of flunixin meglumine (1.1 mg/kg, IV, every 24 h), ceftiofur (2.2 mg/kg IM, every 24 h), and oral or parenteral electrolyte fluids.

### 2.1. Sample Collection and Initial Processing

Whole blood, milk, and urine samples were collected on-farm immediately before euthanasia, placed on ice, and transported to the laboratory for processing within 2 h. Euthanasia was performed by gunshot method in accordance with the American Veterinary Medical Association guidelines [18]. Blood was collected into serum separator and anticoagulant (EDTA) containing tubes, whereas milk and urine samples were collected into tubes with no additives. Milk samples from cows with coliform mastitis were collected only from the infected quarters whereas in control cows, milk was pooled from all quarters. Plasma and serum were harvested by centrifugation (711× *g*, 10 °C, 15 min). Serum and whole blood were submitted for serum biochemistry and complete blood count (CBC) profiles, respectively. Serum and milk samples for antioxidant potential (AOP) and reactive metabolites quantification were immediately flash-frozen in liquid nitrogen. Plasma and milk samples, used for analyses of oxylipids by liquid chromatography and tandem mass spectrometry (LC-MS/MS), were mixed with an antioxidant reducing (AOR) mixture at 4 µL/mL of sample and flash-frozen in liquid nitrogen. The AOR mixture was made up of 0.9 mM butylated hydroxytoluene, 0.54 mM EDTA, 3.2 mM triphenylphosphine, and 5.6 mM indomethacin. Kidney, liver, and mammary gland tissues were cut into small pieces, placed in 1 mL microcentrifuge tubes, and flash-frozen in liquid nitrogen. Other sections from the same tissues were collected into cassettes and placed into 10% formalin for fixation. Mammary tissue samples from cows with coliform mastitis were collected from inflamed parts of infected quarters whereas in control cows, samples were collected from corresponding quarters (front or back). All flash-frozen samples (plasma, milk, serum, and tissues) were stored at −80 °C for later analyses.

### 2.2. Sample Analyses

#### 2.2.1. CBC, Serum Chemistry, and Oxidant Status

Complete blood count and fibrinogen analysis (EDTA whole blood) and serum biochemistry profiles were analyzed at the Michigan State University Veterinary Diagnostic Laboratory (Lansing, MI, USA). The CBC and serum biochemistry were analyzed to demonstrate the clinicopathological abnormalities that develop in cows with severe coliform mastitis relative to healthy controls [19]. For determination of oxidant status, reactive metabolites and the AOP of serum and milk samples were evaluated as previously described [5]. Reactive metabolites were quantified using a commercial assay (Cell Biolabs, San Diego, CA, USA) which determines the florescence signal of a proprietary probe (DCFH-DiOxyQ, Cell Biolabs, San Diego, CA, USA) following its oxidation by reactive metabolites in the sample. The AOP was determined using a colorimetric assay that measures the color intensity of a chemically generated probe. The probe is generated through an oxidation reaction of 2,2-azinobis-(3-ethylbenzothiazoline-6-sulfonic acid) (ABTS) with potassium persulfate [20]. The reducing power of the samples was compared to a synthetic antioxidant standard (Trolox). An oxidant status index (OSi) was also determined by calculating the ratio of the reactive metabolites to the AOP to give the oxidant status [21].

#### 2.2.2. Oxylipid Metabolizing Enzymes: COX2 and sEH

*RNA Extraction*: Extraction of RNA from tissues was performed as previously described [22]. Briefly, tissue samples were cut to roughly 40 mg in size and placed into 450 μL of QIAzol Lysis Reagent (Qiagen Sciences, Germantown, MD, USA) and homogenized with a TissueRuptor II (Qiagen Sciences, Germantown, MD, USA). Another 450 μL of QIAzol was added and samples incubated at room temperature for 5 min after which an additional 180 μL of chloroform was added to tubes. The tubes were then shaken for 15 s and incubated at RT for 3 min. Samples were centrifuged for 15 min at 12,000× *g*, 4 °C. Finally, the upper aqueous layer was removed. RNA was extracted according to the Qiagen RNeasy protocol with a DNase digest step utilizing a QIAcube system (Qiagen, Germantown, MD, USA). Quantification of RNA was carried out using a Take3 Micro-Volume Plate (BioTek, Winooski, VT, USA) on a Synergy H1 microplate reader (BioTek, Winooski, VT, USA).

*Reverse Transcription*: Generation of cDNA was performed as described previously [22]. Briefly, all samples had RNA concentrations adjusted to the same value through dilution with nuclease-free water. A master mix consisting of the high-capacity cDNA reverse-transcription kit (Applied Biosystems, Pleasanton, CA, USA) was added at an equal volume of RNA diluent. Samples were placed into a PTC-200 Peltier Thermo Cycler (MJ Research, Waltham, MA, USA), and ran stepwise as follows: stage 1, 25 °C for 10 min; stage 2, 37 °C for 2 h; stage 3, 85 °C for 5 min; stage 4, hold at 4 °C until removed.

*Real-Time PCR*: Predesigned TaqMan primers with FAM-MGB probes (Applied Biosystems, Pleasanton, CA, USA) were utilized for Real-Time PCR (Table 1). A 7500 Fast Real-time PCR system (Applied Biosystems, Pleasanton, CA, USA) was used for PCR cycling. All genes were assessed as triplicates in a 96-well format with 10 μL per well. Each well contained TaqMan Fast Universal PCR Master Mix (2×) (Applied Biosystems, Vilnius, Lithuania), 20× TaqMan Gene Expression Assay Mix specific to each gene (Applied Biosystems, Pleasanton, CA, USA), sample cDNA (50 ng/well), and nuclease-free water. Thermocycling conditions and data analysis were performed as previously described [22]. Samples were analyzed using DataAssist Software (Version 3.01, Applied Biosystems, Pleasanton, CA, USA) and are presented as 2^−ΔΔCT^ with endogenous controls glucuronidase beta (*GUSB*) and ribosomal protein S9 (*RPS9*). The target genes were prostaglandin synthase 2 (*PTGS2*) and epoxide hydrolase 2 (*EPHX2*), which encode COX2 and sEH enzymes, respectively.

#### 2.2.3. sEH Activity—Ex Vivo Metabolism

Frozen tissue samples were thawed on ice. After thawing on ice, the samples (approximately 5 g each) were mixed with 20 mL of chilled sodium phosphate buffer (20 mM pH 7.4) containing 5 mM EDTA, 1 mM dithiothreitol, and 1 mM phenylmethylsulfonyl fluoride. The mixtures were homogenized with a polytron (Pro Scientific, Oxford, CT, USA), 3-time 30 s pulse at 11,200× *g*. The homogenates were centrifuged at 100,000× g for 60 min at 4 °C. The supernatant (cytosolic fraction) was separated from the pellet, aliquoted, and flash-frozen, then kept frozen at −80 °C until usage. Protein concentration was quantified using the Pierce BCA assay (Pierce, Rockford, IL, USA), using Fraction V bovine serum albumin as the calibrating standard. The residual sEH activity was measured using [^3^H]-*trans*-diphenyl-propene oxide (t-DPPO) as substrate [23]. Briefly, 1 µL of a 5 mM solution of t-DPPO in DMSO was added to 100 µL of diluted homogenate for a final substrate concentration ([S]_final_) of 50 µM. The mixture was incubated at 37 °C for 7 min for liver and kidney or 30 min for mammary gland tissue. The reaction was quenched by adding 60 µL of methanol and 200 µL of isooctane, extracting the remaining epoxide from the aqueous phase. Extractions of the stopped reaction with 1-hexanol were performed in parallel to assess the possible presence of glutathione transferase activity which could also transform the substrate [23]. The activity was followed by measuring the quantity of radioactive diol formed in the aqueous phase using a scintillation counter (TriCarb 2810 TR, Perkin Elmer, Shelton, CT, USA). Assays were performed in triplicate.

#### 2.2.4. Oxylipids

*Sample processing*: Plasma and urine samples previously stored at −80 °C were thawed on ice. One mL of both 4% formic acid, 1 mL plasma sample, 20 µL of AOR agent, and 15 µL of internal oxylipid standards (Cayman Chemical, Ann Arbor, MI, USA) were combined and mixed in glass tubes. Samples were then passed through SPE columns (Oasis HLB 12cc 500 mg LP Extraction Cartridges (Waters, Milford, MA, USA) that had been preconditioned with 12 mL of methanol followed by 12 mL of water. Samples, transferred to the glycerol-containing glass tube, were dried in a Speed Vac (ThermoQuest, Holbrook, NY, USA) at 45 °C heat for 105 min and at least 2 h run-time or until dried. Samples were left to cool to room temperature before the residues were resuspended in 150 µL of a 2:1 methanol and water mixture and set on ice. Samples were transferred into tubes with 0.2 µm bio-inert filters (Millipore, Tullagreen, Ireland) and centrifuged at 14,000× *g* for 30 s to remove residual particulate material. Samples were transferred to chromatography vials with a 150 µL insert and stored at −20 °C until mass spectrometric analysis within 1 week. Milk samples were processed differently with a hydrolyzation step included to improve detection and quantification of oxylipids. Milk samples (0.5 mL) were mixed with 20 µL of AOR and 355 µL of 6 M potassium hydroxide and vortexed briefly to mix. The mixture was then incubated in a water bath at 45 °C for 45 min. After cooling to room temperature, sample mixtures were combined with 1.5 mL of 1% formic acid in acetonitrile, vortexed briefly, and centrifuged at 7359× *g* for 10 min at 4 °C. After centrifugation, the supernatant was carefully removed and transferred to a 50 mL conical tube containing 20 mL of HPLC grade water, and 15 µL of internal standards were added. Samples were then passed through SPE columns (Oasis HLB 12 cc 500 mg LP Extraction Cartridges (Waters, Milford, MA, USA) that had been preconditioned with 12 mL of methanol followed by 12 mL of water. Columns were washed with 10 mL of 5% methanol and eluted into 16 mm × 100 mm glass tubes with 10 µL of 20% glycerol added. Elution was achieved by adding 6 mL of a 9:1 acetonitrile: methanol mixture and dried in a SpeedVac. Dried samples were processed and stored at −80 °C until mass spectrometric analysis like plasma samples.

*LC-MS/MS*: The LC-MS/MS analyses of all plasma and milk samples were performed using a previously reported method [7]. The quantification of oxylipids derived from the CYP450 was accomplished on a Waters Acquity Ultra Performance Liquid Chromatography coupled to a Waters Xevo TQ-S triple quadrupole mass spectrometer (Waters, Milford, MA, USA) using multiple reaction monitoring. Chromatography separation was performed with an Ascentis Express C18 HPLC column, 10 cm × 2.1 mm, 2.7 µm (Supelco, Bellefonte, PA, USA) held at 50 °C. The autosampler was held at 10 °C. Mobile phase A was water with 0.1% formic acid, and mobile phase B was acetonitrile. The flow rate was fixed at 0.3 mL/min. Liquid chromatography separation took 15 min with linear gradient steps programmed as follows (A:B ratio): time 0 to 0.5 min (99:1), to (60:40) at 2.0 min; to (20:80) at 8.0 min; to (1:99) at 9.0 min; 0.5 min held at (1:99) until min 13.0; then return to (99:1) at 13.01 min, and held at this condition until 15.0 min. All oxylipids and fatty acids were detected using electrospray ionization in negative-ion mode. Cone voltages and collision voltages were optimized for each analyte using Waters QuanOptimize software (4.0, Waters, Milford, MA, USA), as reported previously [7]. Ratios of epoxy fatty acids and their sEH-mediated hydrated diols were calculated as an index of sEH activity, as previously reported [24].

#### 2.2.5. Tissue Immunohistochemistry

The 10% neutral-buffered formalin-fixed tissues (kidney, liver, mammary gland) were dehydrated, cleared, and infiltrated with paraffin on the VIP 2000 (Sakura, Torrance, CA, USA). Sections were placed on charged slides and dried at 56 °C overnight. Subsequently, samples were de-paraffinized, hydrated with distilled water, and followed by Tris-buffered saline (TBS) pH 7.4 (Scytek Labs, Logan, UT, USA) for 5 min for pH adjustment. Heat-Induced Epitope Retrieval in Tris-EDTA pH 9.0 was performed using Dako Pascal Micro-Processor controlled pressure cooker (Agilent, Santa Clara, USA) for 30 s at 125 °C and 10 s at 90 °C. Endogenous peroxidase was blocked in a 3% hydrogen peroxide/methanol bath followed by running tap water and distilled water rinses, followed by TBS + Tween 20 (Scytek, Logan, UT, USA) for 5 min. Following pretreatment, standard micro-polymer staining was performed at room temperature on the Biocare intelliPATH™ automated stainer (Biocare, Pacheco, CA, USA). All staining steps followed by rinses in TBS auto wash buffer (Biocare, Pacheco, CA, USA). Non-specific protein blocked using IP FLX Background Punisher (Biocare, Pacheco, CA, USA) for 5 min; Rabbit Polyclonal anti-EPHX2 diluted 1:500 (LS-B6940) (Lifespan Biosciences, Seattle, WA, USA) in standard antibody diluent (Scytek, Logan, UT, USA) and incubated for 1 h; followed by Rabbit on Farma HRP-Polymer™ detection (Biocare, Pacheco, CA, USA) for 30 min. The reaction was developed with Betazoid DAB (Biocare, Pacheco, CA, USA) for 5 min, followed by enhancement with DAB Sparkle (Biocare, Pacheco, CA, USA) for 1 min. Counterstained with CATHE Hematoxylin diluted 1:5 (Biocare, Pacheco, CA, USA) for 5 min, dehydrated, cleared, and mounted with synthetic mounting media. Brightfield images were collected from 10 randomly selected fields on a Nikon Ni Eclipse upright microscope (Nikon Instruments Inc., Tokyo, Japan) using a 20× Plan Apo (NA 0.75) objective and configured with a D2-U3 Nikon Digital Sight color camera (Nikon Instruments Inc., Tokyo, Japan). The images were captured and analyzed using Nikon Elements AR software (version 5.20.01, Nikon Instruments Inc., Tokyo, Japan).

## 3. Statistical Analyses

Statistical analyses were performed using the SAS software (9.4, SAS Institute Inc., Cary, NC, USA). Data were checked for normality by the modified Levene test. Most of the variables were not normally distributed. Consequently, all data were analyzed using the non-parametric tests for unpaired samples using the Wilcoxon rank-sum procedure. Variables compared were sEH activity variables (ex vivo tissue tDPPO metabolism; oxylipid concentrations and their ratios), oxidant status variables (reactive metabolites, AOP, OSi), mRNA expression (COX2, sEH), tissue IHC staining intensity (sEH), and the CBC and serum biochemistry parameters. Statistical significance was declared at *p* ≤ 0.05.

## 4. Results and Discussion

Relative to reference data, CBC and serum biochemistry profiles showed several changes in indicators of acute inflammation in cows with coliform mastitis compared to healthy control cows (Table S1, supplementary data). The white blood cell count was predominantly characterized by neutropenia with increased immature neutrophils in cows with coliform mastitis whereas only one control cow had a slight neutrophilia. The acute phase protein, fibrinogen, which increases with acute inflammation, was elevated above reference range in cows with coliform mastitis and significantly greater than control cows (*p* = 0.008). Serum biochemistry abnormalities were mostly detected in cows with coliform mastitis compared to control cows (Table S1, supplementary data). The CBC and serum biochemistry results mirror previous observations [19] and provided support of an acute inflammation model of cows with coliform mastitis to assess sEH activity and oxidant status.

### 4.1. Mammary Tissue sEH Activity Is Increased during Coliform Mastitis

**Figure 1:**
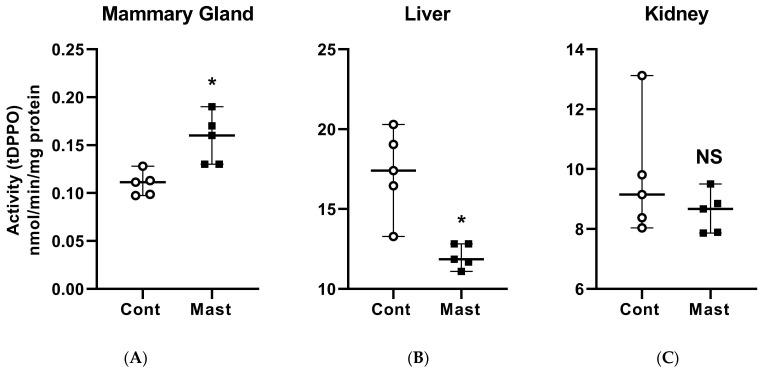
Soluble epoxide hydrolase enzymatic activity based on the metabolism of [^3^H]-*trans*-diphenyl-propene oxide (t-DPPO) (median and range) in the mammary gland (**A**), liver (**B**), and kidney (**C**) tissues from healthy control (Cont) dairy cows (open circles, *n* = 5) and those with systemic coliform mastitis (Mast) (filled black squares, *n* = 5). Data were analyzed with Wilcoxon rank-sum tests (α = 0.05). * *p* < 0.05, NS—not significant.

The ex vivo approach used in this study was validated previously using multiple substrates, including tDPPO, with samples from several animal models of inflammation, including laminitis and lipopolysaccharide-induced synovitis in horses [23,25,26]. Thus, we chose this ex vivo method as a primary outcome measure for assessing the activity of sEH with the subsequent goal of evaluating the modulation of this enzyme in the treatment of bovine coliform mastitis. Mammary gland tissues from cows with mastitis had greater (*p* = 0.008) metabolism of tDPPO, indicating greater sEH activity than control cows (Figure 1). In contrast, the metabolism of tDPPO by liver tissues from cows with coliform mastitis was lower (*p* = 0.008) than from control cows, while metabolism by kidney tissues was not different (*p* = 0.310) between the groups (Figure 1). The activity in the liver and kidney was unexpected because both gene expression and protein concentration of sEH are highest in these tissues in other species [27]. In cattle, mRNA expression of sEH shows a similar greater basal expression in the liver and kidney than other major organs [22]. The contrasting results of sEH activity in tissues could suggest differential tissue-specific regulation. Gene expression data (CYP450, sEH) and sEH inhibition from other acute inflammatory models are contradictory with acute decreases or increases in some studies [28,29]. Despite this discrepancy, mortality rates and clinical signs were improved by chemical inhibition of sEH or its gene deletion in the same models [12,30], underscoring the potential of sEH as a target for therapy in acute inflammation.

### 4.2. Prediction of sEH Activity by Epoxy Fatty Acid to Vicinal Diols during Coliform Mastitis

The activity of sEH was also assessed indirectly by calculating epoxy fatty acid/vicinal diol ratios to characterize the in vivo activity [31]. Four epoxy fatty acid isomers (5,6-; 8,9-; 11,12-; 14,15 epoxyeicosatrienoic acids (EETs)) generated from arachidonic acid (ArA) are metabolized by sEH to corresponding vicinal diol isomers (5,6-; 8,9-; 11,12-; 14,15 dihydroxyepoxyeicosatrienoic acids (DHETs)) while two epoxy fatty acid isomers (9,10- and 12,13- epoxyoctadecenoic acid (EpOME)) from Linoleic acid (LA) are correspondingly metabolized to [9,10- and 12,13-dihydroxy epoxyoctadecenoic acid (DiHOME)] [14]. Comparatively, larger ratios suggest a greater accumulation of the upstream epoxides and or a lesser concentration of the vicinal diols generated by sEH activity. Smaller ratios suggest lower epoxide and or greater vicinal diol concentrations. Our study showed that metabolite ratios do not necessarily match with direct sEH activity determined by the ex vivo metabolism of tDPPO. Concentrations of both ArA- and LA-derived epoxy fatty acids (sEH substrates) in milk did not differ between mastitis and control cows (Table 2). Cows with coliform mastitis had greater ArA-derived epoxides in plasma but similar in urine compared to controls. In contrast, cows with mastitis had lower LA-derived epoxide concentrations in both plasma and urine than controls. (Table 2). The mean concentration of one ArA-derived epoxide (14,15-EET) was greater in plasma from cows with mastitis, which was opposite to the lower concentrations of both LA-derived epoxides (9,10- and 12,13-EpOME) relative to control cows (Table 2).

The LA-derived vicinal diols were lower in milk from cows with mastitis (*p* = 0.02) than controls (Table 3). Of the diols measured in plasma, only 9,10-DiHOME, a hydration product of 9,10-EpOME, was lower (*p* = 0.03) in plasma from cows with mastitis compared to controls (Table 3).

The corresponding ratios of the epoxy fatty acids to DiHOMEs in milk were lower (*p* < 0.05) in milk from cows with mastitis compared to healthy controls (Table 4). Despite the lack of differences in the concentrations of 8,9-EET or 8,9-DHETs, the ratio of these ArA-derived metabolites in milk was also lower (*p* = 0.04) in cows with mastitis compared to control (Table 4). For ArA-derived metabolites, the plasma metabolite ratio in cows with mastitis was greater (*p* < 0.05) for the 11,12-EET/11,12-DHET pair relative to the healthy control cows (Table 4). Conversely, the plasma ratio of the LA-derived metabolites (9,10-EpOME/9,10-DiHOME) was lower (*p* = 0.0002) in cows with mastitis compared to healthy cows (Table 4).

From these results, metabolite ratios in milk predicted lower sEH activity in coliform mastitis cows which contrasted the ex vivo metabolism of tDPPO. In plasma, predicted sEH activity based on ratios was dependent on the PUFA substrate. When compared to ex vivo metabolism of tDPPO in mammary tissue, the ratios from ArA-derived oxylipids indicated decreased sEH activity, whereas ratios from LA-derived oxylipids increased sEH. Several possibilities could explain the disagreement between the complementary approaches for evaluating sEH activity. The treatment of all cows with flunixin meglumine, which blocks COX-mediated oxylipid production, may have impacted concentrations of oxylipids from other pathways, including the CYP450 and the sEH pathways. Effects of COX2 blockade on epoxy fatty acid and vicinal diol production are not entirely known; however, administration of specific COX2 blockers in mice increases 20-hydroxyeicosatetraenoic acid [32], an oxylipid produced by the CYP450 pathway. The utility of metabolite ratios to predict sEH activity should be evaluated before treatment interventions that can affect oxylipid production like NSAIDs.

The lack of agreement between metabolite ratios and tDPPO metabolism may be related to the several fates of oxylipids which include cell membrane re-esterification [33,34], conjugation and excretion [35] possible metabolism through other pathways that may be activated during acute inflammation (COX, ROS-mediated) [14,33,36]. Other fates of epoxides and vicinal diols, including chain elongation, beta-oxidation, omega oxidation, and reincorporated into phospholipid membranes, may differentially affect their measured concentrations [37]. As such, sample processing must be designed to account for the various fates of oxylipids, such as hydrolysis of samples, to free esterified or conjugated oxylipids [35]. Epoxy fatty acids and vicinal diols are preferentially metabolized. For example, LA-derived metabolites are more likely to be esterified and preferentially metabolized than ArA-derived [38,39], which may explain their opposing prediction of sEH activity in plasma. The use of oxylipid concentrations in fluid samples must also be compared to oxylipids quantified from tissues to determine which metabolite concentrations correlate among different samples. In a murine inflammatory model, oxylipid concentrations and changes in tissues differed from plasma [40]. Improved and optimized sample processing, extraction, and quantification procedures are needed for detecting epoxides and their vicinal diols to improve the use of these metabolites for in vivo sEH activity assessment in cattle.

### 4.3. Oxidant Status Is Increased in the Mammary Gland of Coliform Mastitis Cows

Oxidative stress increases sEH expression and activity by activating the redox-sensitive transcriptional factor AP-1 [41]. Bovine coliform mastitis is associated with oxidative stress [5]. Therefore, we quantified changes in oxidant status to show alterations in oxidant status at the time when sEH activity was determined. Measures of oxidant status showed a lower AOP and a greater reactive metabolite concentration in milk from cows with coliform mastitis (Table 5). The OSi was greater in milk from cows with mastitis compared to controls, suggesting a greater prooxidant status. In serum, both AOP and reactive metabolites were at lower concentrations in cows with mastitis (*p* < 0.05). The serum OSi in cows with coliform mastitis was lower than in serum from healthy control cows, suggesting a lower prooxidant status. (Table 5).

**Figure 2:**
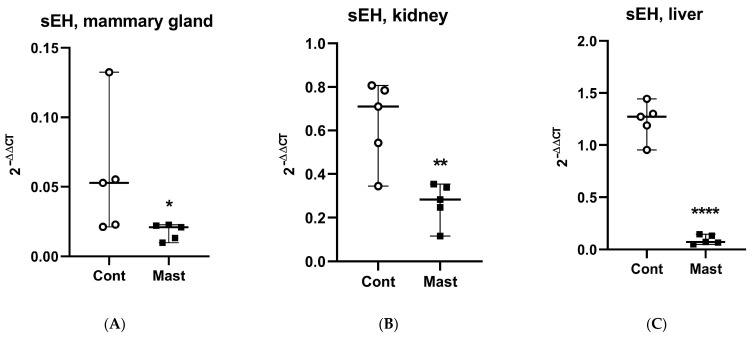
Relative expression of soluble epoxide hydrolase (sEH) mRNA (median and range) in tissues: (**A**) mammary gland, (**B**) kidney, (**C**) liver from dairy cows with systemic coliform mastitis (*n* = 5) and matched control dairy cows with no overt clinical disease (*n* = 5). Data were analyzed with Wilcoxon rank-sum tests (α = 0.05). * *p* < 0.05, ** *p* < 0.01, **** *p* < 0.0001.

**Figure 3:**
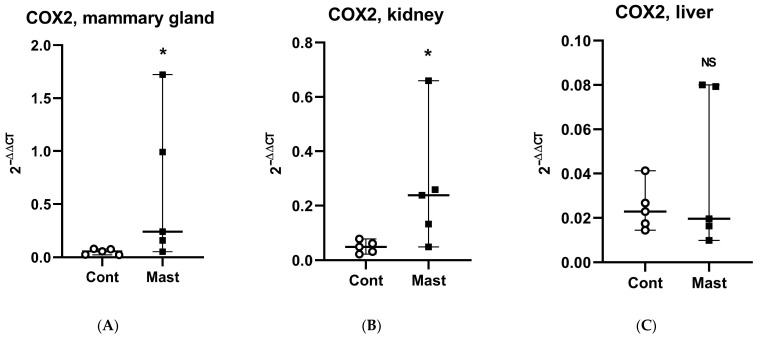
Relative expression of cyclooxygenase (COX2) mRNA (median and range) in tissues (**A**) mammary gland, (**B**) kidney, (**C**) liver from dairy cows with systemic coliform mastitis (*n* = 5) and matched control dairy cows with no overt clinical disease (*n* = 5). Data were analyzed with Wilcoxon rank-sum tests (α = 0.05). * *p* < 0.05, NS—not significant.

Although oxidant status was not determined in tissues where the direct sEH activity (tDPPO metabolism) was measured, it is interesting to note the greater prooxidant status in milk and the greater mammary sEH activity in cows with coliform mastitis. Other studies linked mammary tissue gene expression changes to changes in oxidant status parameters in milk [42]. Conversely, the lower prooxidant status in plasma and the lower sEH activity in the liver are interesting observations. The possibility exists that the oxidant status was related to the sEH activities in these tissues based on the stimulatory effect of oxidative stress on sEH gene expression [43]. Despite this potential, our data show that across all tissues examined (mammary, liver, and kidney), mRNA expression for sEH was lower in cows with mastitis compared to healthy controls. Thus, gene expression may not explain the greater metabolism of tDPPO in the mammary tissues from cows with coliform mastitis. It is interesting to note that basal expression of sEH in the mammary gland was among the lowest relative to the highest expression in the liver [22]. In contrast to sEH, mRNA expression for the proinflammatory marker, COX2, was increased in mammary and kidney tissues (Figure 2 and Figure 3).

**Figure 4:**
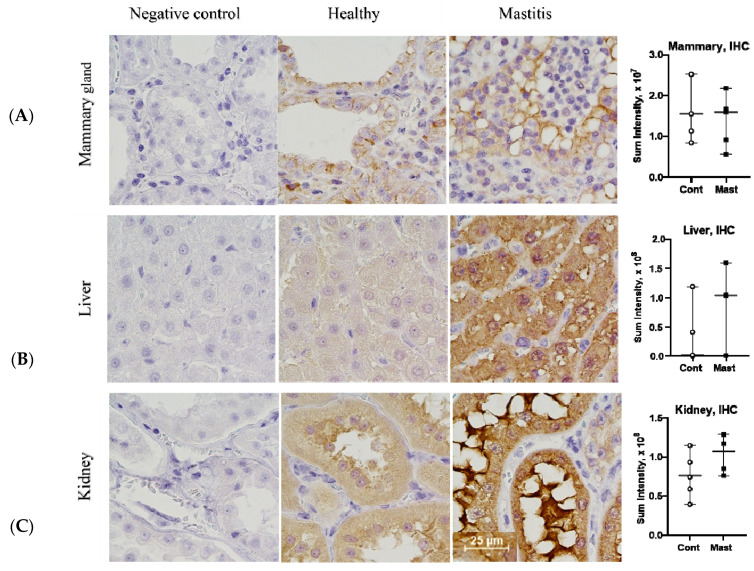
IHC staining intensity for soluble epoxide hydrolase protein (Median and Range, graphs) in the mammary gland (**A**), liver (**B**), kidney (**C**) in cows with mastitis (Mast) and healthy controls (Cont) (1 representative mastitis case and a matched control). IHC, immunohistochemistry, bar—25 µm.

The mRNA expression agrees with observations in acute inflammatory murine models where in vivo depression of CYP450 isoforms and sEH gene expression was found in the various organs, including the liver and kidneys [28]. We examined protein abundance in tissues through IHC staining to determine if levels of sEH activity based on ex vivo tDPPO metabolism could be explained by protein abundance. There were no differences (*p* > 0.05) in the IHC sEH staining intensity in tissues between cows with mastitis and controls (Figure 4), suggesting differences in activity may be explained by posttranslational rather than transcriptional regulation of sEH. Possible posttranslational regulation of sEH may include suicidal inactivation, which occurs in other oxylipid synthesizing enzymes like LOX, COX, and thromboxane A2 synthase [44,45,46]. Different oxylipids may also modify the activity of sEH and reduce its activity. For example, 15-deoxy-prostaglandin J2 (15d-PGJ2) inhibits allosterically through binding at a non-catalytic site of the sEH enzyme [47]. Although oxylipids from other pathways are not reported in this study, increased COX2 mRNA expression could have contributed to the formation of prostaglandin D from which 15d-PGJ2 is formed. Our previous studies showed that 15d-PGJ2 was only found in cows with coliform mastitis [7]. Further studies should characterize mechanisms that contribute to modifying sEH activity and evaluating different approaches modulating its activity.

The specific relationship between oxidant status and sEH activity during coliform mastitis cannot be determined from our study because samples were collected only at the terminal stages of clinical disease. Our findings may not reflect cows in the early stages of the clinical disease when interventions may be beneficial. The decreased plasma OSi in cows with coliform mastitis differs from sepsis studies in humans that showed negative outcomes associated with progressive prooxidant production [48]. Skeletal muscle damage in human sepsis contributes to prooxidant production and pathology [49]. In cows with coliform mastitis in our study, serum biochemistry findings (increased creatinine kinase and aspartate aminotransferase enzymes) were suggestive of muscle damage (supplementary Table S1), yet the plasma OSi was lower. Treatment interventions in cows with coliform mastitis may explain, in part, the lower plasma OSi. Resuscitation fluids cause dilution and redistribution of antioxidants to tissues in sepsis [48] and could potentially impact concentrations of prooxidants. The blockade of COX2 may also contribute to the lower plasma prooxidant concentrations by decreasing the formation of ROS and oxylipids [6]. Further analysis of changes in the OSi during bovine coliform mastitis is needed to design appropriate therapies to better address oxidative stress and improve clinical disease outcomes.

In summary, we report, for the first time, a greater sEH activity in the mammary gland tissues during systemic bovine coliform mastitis at the same time when prooxidant production is increased in the mammary gland based on milk samples. The decreased hepatic and renal sEH activity in the cows of this study may suggest that terminal stages of the disease may not respond to therapy. Characterizing hepatic and renal sEH activities and tissue oxidant status require further investigation to determine optimal intervention strategies. We also report that based on the current methods of quantification and analyses, the oxylipid substrates and hydration products of sEH quantified in milk do not predict the activity of sEH in tissue as evaluated by ex vivo analysis. We, therefore, propose further exploration of the sEH activity patterns and how they correlate with changes in oxidant status to determine if targeting sEH modulates oxidative stress.

## 5. Conclusions

In conclusion, we found that sEH activity is increased in mammary tissue but decreased in the liver and kidneys in cows at the terminal stages of systemic bovine coliform mastitis. The increased sEH activity in the mammary gland at the same time with the increased prooxidant status in milk during coliform mastitis may suggest a possible relationship between oxidative stress and the sEH activity. This finding may mean that therapies targeting the sEH activity may need to be targeted to the mammary gland, but this needs further exploration.

## Figures and Tables

**Table 1:** Proprietary TaqMan primer reference information.

Gene	NCBI Reference Sequence ^1^	TaqMan Assay ID
*PTGS2*	NM_174445.2	Bt03214492_m1
*EPHX2*	NM_001075534.1	Bt03241449_m1
*GUSB*	NM_001083436.1	Bt03256165_m1
*RPS9*	NM_001101152.2	Bt03272017_m1

^1^ National Center for Biotechnology Information reference sequence found in the nucleotide database (https://www.ncbi.nlm.nih.gov/nuccore/, accessed on 4 June 2018). *PTGS2*, prostaglandin synthase 2; *EPHX2*, epoxide hydrolase 2; *GUSB*, glucuronidase beta; *RPS9*, ribosomal protein S9.

**Table 2:** Cytochrome P450-derived epoxy fatty acids (sEH substrates) in milk, plasma, and urine of systemic coliform mastitis and healthy control cows (median and range, *n* = 5/group).

Sample	* Metabolites	PUFA Substrate	Mastitis	Control	*p*-Value
Milk	8,9-EET	ArA	10.70 (7.1–55.80)	1.4 (0.80–2.00)	NS
	11,12-EET	ArA	3.20 (1.80–9.80)	1.50 (1.10–2.50)	0.06
	14,15-EET	ArA	3.30 (0.40–8.60)	1.70 (1.10–3.10)	NS
	9,10-EpOME	LA	122.00 (88.00–268.00)	100.00 (58.00–138.00)	NS
	12,13-EpOME	LA	62.00 (30.00–210.00)	84.00 (48.00–114.00)	NS
Plasma	8,9-EET	ArA	0.03 (0.03–0.10)	0.03 (0.03–0.10)	NS
	11,12-EET	ArA	0.30 (0.01–0.10)	0.01 (0.01–0.10)	NS
	14,15-EET	ArA	0.10 (0.10–0.30)	0.01 (0.01–0.10)	0.02
	9,10-EpOME	LA	2.00 (0.20–8.00)	8.00 (6.00–8.00)	0.02
	12,13-EpOME	LA	2.00 (0.20–2.00)	8.00 (4.00–8.00)	0.0003
Urine	8,9-EET	ArA	0.03 (0.03–0.20)	0.03 (0.03–0.10)	NS
	11,12-EET	ArA	0.01 (0.01–0.10)	0.01 (0.01–0.01)	NS
	14,15-EET	ArA	0.01 (0.01–0.01)	0.01 (0.01–0.01)	-
	9,10-EpOME	LA	0.20 (0.20–6.00)	0.20 (0.20–0.20)	NS
	12,13-EpOME	LA	2.00 (2.00–2.00)	2.000 (0.20–2.00)	NS

* Metabolite concentrations are nM; EET, epoxyeicosatrienoic acid; DHET, dihydroxyepoxyeicosatrienoic acid; EpOME, epoxyoctadecenoic acid; DiHOME, dihydroxyepoxyoctadecenoic acid; ArA, arachidonic acid; LA, linoleic acid; PUFA, polyunsaturated fatty acid; Data were analyzed with Wilcoxon rank-sum tests (α = 0.05). NS—not significant.

**Table 3:** Soluble epoxide hydrolase-derived (dihydroxy) epoxy fatty acids in milk, plasma, and urine of systemic coliform mastitis and healthy control cows (median and range, *n* = 5/group).

Sample	* Metabolites	PUFA Substrate	Mastitis	Control	*p*-Value
Milk	8,9-DHET	ArA	1.80 (0.80–14.70)	1.20 (0.40–2.10)	NS
	11,12-DHET	ArA	2.90 (1.10–24.80)	2.70 (1.00–5.60)	NS
	14,15-DHET	ArA	2.70 (0.10–3.80)	3.90 (0.40–12.30)	NS
	9,10-DiHOME	LA	125.00 (43.00–204.00)	569.00 (175.00–1081.00)	0.02
	12,13-DiHOME	LA	55.00 (25.00–114.00)	379.00 (98.00–689.00)	0.02
Plasma	8,9-DHET	ArA	0.50 (0.30–1.50)	0.70 (0.60–2.10)	NS
	11,12-DHET	ArA	1.90 (0.30–5.50)	2.90 (2.50–3.00)	NS
	14,15-DHET	ArA	3.50 (0.20–6.60)	1.60 (0.70–4.10)	NS
	9,10-DiHOME	LA	8.00 (6.00–39.00)	32.00 (30.00–76.00)	0.03
	12,13-DiHOME	LA	0.10 (0.10–1.00)	0.10 (0.10–0.10)	NS
Urine	8,9-DHET	ArA	0.10 (0.02–0.60)	0.10 (0.02–0.40)	NS
	11,12-DHET	ArA	0.03 (0.03–3.50)	0.10 (0.03–0.20)	NS
	14,15-DHET	ArA	2.70 (0.60–4.10)	5.60 (1.00–9.00)	NS
	9,10-DiHOME	LA	6.00 (2.00–11.00)	2.00 (2.00–5.00)	NS
	12,13-DiHOME	LA	0.10 (0.10–5.00)	0.10 (0.10–1.00)	NS

* Metabolite concentrations are nM; EET, epoxyeicosatrienoic acid; EpOME, epoxyoctadecenoic acid; DHET, dihydroxyepoxyeicosatrienoic acid; DiHOME, dihydroxyepoxyoctadecenoic acid; ArA, arachidonic acid; LA, linoleic acid; PUFA, polyunsaturated fatty acid; Data were analyzed with Wilcoxon rank-sum tests (α = 0.05). NS—not significant.

**Table 4:** Ratio epoxides to their sEH-derived vicinal diols in milk, plasma, and urine of systemic coliform mastitis and healthy control cows (median and range, *n* = 5/group).

Sample	Metabolite Ratios	PUFA Substrate	Mastitis	Control	*p*-Value
Milk	8,9-EET/8,9-DHET	ArA	3.94 (1.53–10.75)	0.95 (0.70–2.75)	0.04
	11,12-EET/11,12-DHET	ArA	0.90 (0.13–8.91)	0.42 (0.27–1.40)	NS
	14,15-EET/14,15-DHET	ArA	1.89 (0.80–4.00)	0.72 (0.14–3.75)	NS
	9,10-EpOME/9,10-DiHOME	LA	1.78 (0.43–2.42)	0.15 (0.10–0.34)	0.006
	12,13-EpOME/12,13-DiHOME	LA	1.84 (0.35–2.48)	0.24 (0.12–0.49)	0.01
Plasma	8,9-EET/8,9-DHET	ArA	0.06 (0.04–0.10)	0.05 (0.03–0.05)	0.06
	11,12-EET/11,12-DHET	ArA	0.13 (0.03–0.20)	0.004 (0.003–0.033)	0.004
	14,15-EET/14,15-DHET	ArA	0.06 (0.02–1.50)	0.006 (0.002–0.143)	NS
	9,10-EpOME/9,10-DiHOME	LA	0.33 (0.01–0.57)	0.19 (0.11–0.25)	NS
	12,13-EpOME/12,13-DiHOME	LA	2.00 (2.00–20.00)	80.00 (40.00–80.00)	0.0002
Urine	8,9-DHET/8,9-DHET	ArA	0.30 (0.05–2.00)	0.30 (0.08–1.50)	NS
	11,12-EET/11,12-DHET	ArA	0.33 (0.03–0.33)	0.10 (0.05–0.33)	NS
	14,15-EET/14,15-DHET	ArA	0.004 (0.002–0.170)	0.002 (0.001–0.010)	NS
	9,10-EpOME/9,10-DiHOME	LA	0.10 (0.03–0.55)	0.10 (0.04–0.10)	NS
	12,13-EpOME/12,13-DiHOME	LA	20.00 (0.40–20.00)	20.00 (2.00–20.00)	NS

EET, epoxyeicosatrienoic acid; EpOME, epoxyoctadecenoic acid; DHET, dihydroxyepoxyeicosatrienoic acid; DiHOME, dihydroxyepoxyoctadecenoic acid; ArA, arachidonic acid; LA, linoleic acid; PUFA, polyunsaturated fatty acid; Data were analyzed with Wilcoxon rank-sum tests (α = 0.05). NS—not significant.

**Table 5:** Serum and milk reactive metabolites, antioxidant potential (AOP), and oxidant status index (OSi) in dairy cows with systemic mastitis and matched healthy controls (median and range, *n* = 5).

Sample	Parameter	Control	Mastitis	*p* Value
Serum	Reactive metabolites, FU/µL	14.49 (9.27–20.71)	8.38 (4.48–10.60)	0.01
	AOP, TE/ µL	21.35 (20.59–24.95)	19.56 (17.33–21.38)	0.03
	OSi, arbitrary units	0.70 (0.37–0.99)	0.40 (0.23–0.58)	0.04
Milk	Reactive metabolites, FU/µL	5256 (4718–6608)	14,547 (8194–32224)	0.03
	AOP, TE/ µL	17.56 (17.10–20.27)	15.29 (11.58–16.85)	0.01
	OSi, arbitrary units	278.6 (248.4–386.4)	951.2 (569.0–2669)	0.03

FU, florescence units; AOP, antioxidant potential; TE, Trolox equivalents; OSi, oxidant status index.

## Data Availability

Data generated and analyzed are included in this manuscript.

## Supplementary Materials

The following are available online at https://www.mdpi.com/article/10.3390/antiox10050812/s1, Table S1: Serum biochemistry and complete blood count parameters (median and range) in dairy cows with systemic mastitis and matched healthy controls (*n* = 5/group).
