# Supplementary material for: Activity of sEH and Oxidant Status during Systemic Bovine Coliform Mastitis
Source: Antioxidants (Basel). 2021 May 20;10(5):812. doi: 10.3390/antiox10050812 (PMC8161397; doi:10.3390/antiox10050812)
Supplement: Supplementary file 1 [file antioxidants-10-00812-s001.zip › antioxidants-1218250-supplementary.pdf]

Supplementary Data: Activity of sEH and oxidant status during systemic bovine coliform mastitis. Mavangira et. el.

Table S1. Serum biochemistry and complete blood count parameters (median and range) in dairy cows with systemic mastitis and matched healthy controls (n = 5/group).

Parameter	*Reference Range	Coliform Mastitis Group	Healthy Controls Group	*p*-Value
**Serum Biochemistry Parameters**				
**SUN**	[7–19] mg/dL	32 (21–68)	12 (8–15)	0.008
**Creatinine**	[0.6–1.1] mg/dL	1.3 (0.9–2.2)	0.8 (0.8–0.8)	0.008
**Sodium**	[132–141] mmol/L	140 (125–142)	137 (134–138)	0.127
**Potassium**	[3.6–5.3] mmol/L	3.7 (3–5.4)	4.4 (4–4.7)	0.318
**Chloride**	[92–101] mmol/L	88 (81–99)	96 (94–98)	0.595
**Bicarbonate**	[23–32] mmol/L	29 (10–38)	27 (25–30)	0.579
**NA/K**	[26–38]	38 (23–47)	31 (29–34)	0.246
**Anion Gap**	[12–23] mmol/L	21 (12–36)	17 (17–19)	0.151
**Osmolarity**	[273–290] mmol/L	295 (263–311)	281 (276–285)	0.135
**Calcium**	[8.8–10.4] mg/dL	8.4 (7.4–9)	9.1 (8.5–9.4)	0.024
**Phosphorus**	[4.5–7.3] mg/dL	7.8 (4.6–9.1)	6.1 (5.6–6.4)	0.151
**Magnesium**	[1.9–2.8] mg/dL	2.3 (1.7–2.7)	2.3 (2.2–2.4)	>0.999
**TP - serum**	[6.4–8.1] g/dL	6.6 (5.7–6.8)	7.9 (7.1–8.2)	0.008
**Albumin**	[3.2–3.9] g/dL	2.9 (2.7–3.7)	3.3 (3.1–3.6)	0.191
**Globulin**	[2.9–4.7] g/dL	3.2 (2.8–3.7)	4.6 (3.6–4.9)	0.016
**Glucose**	[54–77] mg/dL	71 (62–109)	73 (64–84)	0.802
**Bilirubin**	[0.1–0.4] mg/dL	1.4 (0.3–5.6)	0.2 (0.2–0.3)	0.016
**ALP**	[26–85] U/L	66 (49–157)	38 (22–48)	0.008
**GGT**	[11–51] U/L	26 (23–39)	21 (18–28)	0.048
**AST**	[47–120] U/L	319 (170–814)	56 (47–66)	0.008
**CK**	[73–346] U/L	4032 (393–32278)	122 (110–185)	0.008
**Cholesterol**	[119–324] mg/dL	134 (85–197)	193 (154–311)	0.056
**TP - plasma**	[7.4–9.2] g/dL	7.5 (7.1–8.5)	8.8 (8.3–10)	0.032
**Fibrinogen**	[0.1–0.5] g/dL	0.9 (0.5–1.3)	0.5 (0.3–0.7)	0.040
**Red and White Blood Cell Parameters**				
**RBC**	[5.3–7.4] x10^6/μL	6.4 (3.2–8.7)	5.8 (5.3–7.6)	0.968
**Hemoglobin**	[9.5–12.2] g/dL	11.4 (5.3–14.5)	10.1 (9.5–11.9)	0.841
**Hematocrit**	[26–34] %	30 (15–37)	27 (24–32)	0.889
**MCV**	[41–53] fL	46 (37–53)	46 (42–50)	>0.999
**MCH**	[15–20] pg	18 (17–18)	17 (16–19)	0.008
**MCHC**	[35–38] g/dL	38 (34–39)	38 (37–39)	0.651
**CHCM**	[34–38] g/dL	37 (33–38)	37 (35–38)	0.738
**RDW**	[16–20] %	17 (17–25)	19 (18–20)	0.548
**Platelet**	[217–444] x10^3/μL	241 (127–552)	366 (80–458)	0.841
**MPV**	fL	7.9 (6.4–8.6)	6.5 (2.2–6.5)	0.056
**WBC**	[5.2–11.8] x10^3/μL	4.9 (1.1–10.4)	6.8 (4.4–21.6)	0.310
**Seg Neut**	[1.9–6.1] x10^3/μL	0.2 (0.1–2.8)	3.2 (2.2–6.5)	0.032
**Band Neut**	[0.0–0.0] x10^3/μL	0.5 (0.1–5.2)	0 (0–0)	0.008
**Lymphocyte**	[1.6–8.3] x10^3/μL	2.2 (0.8–3.9)	2.2 (1.6–13.8)	0.421
**Monocyte**	[0.0–1.0] x10^3/μL	0 (0–0.1)	0.2 (0.2–0.9)	0.008

**Eosinophils**	[0.0–0.9] x10³/μL	0 (0–0.2)	0.2 (0–0.4)	0.206

*Michigan State University Veterinary Diagnostic Laboratory reference ranges; SUN, serum urea nitrogen; NA/K, sodium/potassium ratio; TP, total protein; ALP, alkaline phosphatase; GGT, gamma-glutamyl transferase; AST, Aspartate aminotransferase; CK, creatine kinase; RBC, red blood cell count, MCV, mean corpuscular volume; MCH, mean corpuscular hemoglobin; MCHC, mean corpuscular hemoglobin concentration; CHCM, cellular hemoglobin concentration mean; RDW, red blood cell distribution width; MPV, mean platelet volume; WBC, white blood cell count; Seg Neut, segmental neutrophils; Band Neut, band (immature) neutrophils. Data were analyzed with Wilcoxon rank-sum tests ($\alpha = 0.05$).
